# Stress dynamics that maintain posttraumatic stress disorder across 20 years

**DOI:** 10.1017/S0033291725000686

**Published:** 2025-05-19

**Authors:** Whitney R. Ringwald, Scott Feltman, Sean A.P. Cloutson, Frank Mann, Camilo Ruggero, Evelyn Bromet, Benjamin J. Luft, Roman Kotov

**Affiliations:** 1 Department of Psychology, University of Minnesota, Minneapolis, MN, USA; 2 Department of Applied Mathematics, Stony Brook University, Stony Brook, NY, USA; 3 Program in Public Health and Department of Family, Population, and Preventive Medicine, Renaissance School of Medicine, Stony Brook University, Stony Brook, NY, USA; 4 Department of Medicine, Stony Brook University, Stony Brook, NY, USA; 5 Department of Psychology, University of North Texas, Denton, TX, USA; 6 Department of Psychiatry, Stony Brook University, Stony Brook, NY, USA; 7 World Trade Center Health Program, Stony Brook University, Stony Brook, NY, USA

**Keywords:** life stress, life events, internalizing psychopathology, longitudinal, multilevel structural equation model

## Abstract

**Background:**

Posttraumatic stress disorder (PTSD) is often chronic and impairing. Mechanisms that maintain symptoms remain poorly understood because of heterogenous presentation. We parsed this heterogeneity by examining how individual differences in stress-symptom dynamics relate to the long-term maintenance of PTSD.

**Methods:**

We studied 7,308 trauma-exposed World Trade Center responders who self-reported PTSD symptoms and stressful life events at annual monitoring visits for up to 20 years (average = 8.8 visits; [range = 4–16]). We used multilevel structural equation models to separate the stable and time-varying components of symptoms and stressors. At the within-person level, we modeled stress reactivity by cross-lagged associations between stress and future symptoms, stress generation by cross-lagged associations between symptoms and future stress, and autoregressive effects represented symptom persistence and stress persistence. The clinical utility of the stress-symptom dynamics was evaluated by associations with PTSD chronicity and mental health care use.

**Results:**

Stress reactivity, stress generation, and symptom persistence were significant on average (bs = 0.03–0.16). There were significant individual differences in the strength of each dynamic (interquartile ranges = 0.06–0.12). Correlations among within-person processes showed some dynamics are intertwined (e.g. more reactive people also generate stress in a vicious cycle) and others represent distinct phenotypes (e.g. people are reactive or have persistent symptoms). Initial trauma severity amplified some dynamics. People in the top deciles of most dynamics had clinically significant symptom levels across the monitoring period and their health care cost 6–17× more per year than people at median levels.

**Conclusions:**

Individual differences in stress-symptom dynamics contribute to the chronicity and clinical burden of PTSD.

## Introduction

Many people with posttraumatic stress disorder (PTSD) experience symptoms for years after the initial trauma (Bryant et al., 2015; Galatzer-Levy et al., 2018; Marmar et al., 2015; Solomon & Mikulincer, 2006). Indeed, the majority of people with PTSD have symptoms even after undergoing gold standard treatment (Hoskins et al., 2015; Steenkamp, Litz, & Marmar, 2020; Steenkamp, Litz, Hoge, & Marmar, 2015). This often prolonged course of PTSD, and ongoing need for mental health services, comes at a considerable economic cost to the individual and society (Von Der Warth, Dams, Grochtdreis, & König, 2020). Identifying mechanisms that determine a chronic symptom course is imperative to develop more targeted and effective interventions and to reduce socioeconomic burden. Psychological mechanisms involving transactions between stressful life events and symptoms are among the more promising candidates for explaining the long-term maintenance of PTSD, and they can be directly translated into intervention targets (Chiu, Low, Chan, & Meiser-Stedman, 2024; Hammen, 1991; Monroe & Harkness, 2005). Next, we review four dynamics reflecting mechanisms that have been proposed to help explain PTSD symptoms over time, which we term: (1) Stress Reactivity, (2) Stress Generation, (3) Symptom Persistence, and (4) Stress Persistence.

## Stress-symptom dynamics and underlying maintenance mechanisms PTSD

One candidate dynamic is *stress reactivity*, which refers to how stressful events occurring after the initial trauma can exacerbate symptoms. Stressors may be especially potent for people with PTSD because of pre-trauma vulnerabilities associated with the condition (Brewin, Andrews, & Valentine, 2000; Chiu et al., 2024; Nievergelt et al., 2019). Longitudinal studies of trauma-exposed populations support the role of stress reactivity, with many finding that post-trauma stressors predict subsequent increases in PTSD symptoms and that people exposed to more traumatic experiences are especially reactive (Galea et al., 2008; Lowe et al., 2017; Schmied, Larson, Highfill-McRoy, & Thomsen, 2016). Furthermore, according to stress sensitization models, trauma exposure may actually cause biological, cognitive, and behavioral changes that amplify responses to stressful life events (Post & Weiss, 1998). Some mechanisms that have been proposed to explain heightened reactivity after trauma include abnormalities in neurobiological systems involved with memory (Careaga, Girardi, & Suchecki, 2016; Foa, Steketee, & Rothbaum, 1989), selective attention to threat (Ehlers & Clark, 2000), and engagement in avoidant coping strategies that prevent adaptive fear conditioning (Seligowski, Lee, Bardeen, & Orcutt, 2015). In support of the possibility trauma contributes to stress reactivity, there is also evidence that the more traumatic events a person is exposed to, the more reactive they are to subsequent stressors (Smid et al., 2012, 2013; Zvolensky et al., 2015a). Thus, regardless of whether reactivity emerges before or after a trauma, PTSD pathology may be prolonged by the continual re-activation of symptoms in response to stressful life events.

A second candidate mechanism is *stress generation.* Stress generation refers to how symptoms may actually create more stressful conditions that perpetuate a person’s problems (Hammen, 1991; Rnic et al., 2023). In support of stress generation mechanisms in PTSD, longitudinal studies have shown that PTSD symptoms prospectively predict stressful life events (Lowe et al., 2014; Maniates et al., 2018; Milan, Zona, Acker, & Turcios-Cotto, 2013; Sadeh, Miller, Wolf, & Harkness, 2015; Schmied et al., 2016; Zvolenskyet al., 2015b). In conjunction with stress reactivity, stress generation may perpetuate a feedback loop that prolongs PTSD in which intense reactions to stress create more stress, that lead to more intense reactions and so on.

A third dynamic is *symptom persistence*, which could reflect a slowed return to baseline functioning. Recovery may be slowed because of, for example, inadequate treatment, limited social support, or underdeveloped coping skills (Brewin et al., 2000). Additionally, stress autonomy or ‘kindling’ models propose that neurobiological changes induced by trauma lower a person’s threshold for stress to a point that trauma-related symptoms are sustained in the absence of major life stressors (Monroe & Harkness, 2005; Post et al., 1997). There is longitudinal evidence for this sort of symptom persistence over and above the effects of stressful events in depression (Kendler & Gardner, 2016), but no studies have examined this mechanism in the context of PTSD. Given the common features with depression, it is possible that symptom persistence contributes to the course of PTSD as well.

In addition to the immediate impact of major stressors, being exposed to unremitting stress may contribute to PTSD. *Stress persistence* is therefore a fourth potential mechanism, which encompasses prolonged stressors (e.g. health problems) or cascades of stressful events that unfold over the course of years (e.g. getting an injury leads to job loss which leads to debt). Such conditions of persistent stress may have an indirect effect on the chronicity of PTSD vis-à-vis the toll on a person’s emotional, biological, and material resources (Davidson & Baum, 1986; Hilton et al., 2020).

## Could variation in stress-symptom dynamics govern the long-term course of PTSD?

All longitudinal research on symptoms and stressful events to date has focused on average effects, which ignores the wide variability in the clinical presentation, course, and maintenance mechanisms of PTSD (Bryant, Galatzer-Levy, & Hadzi-Pavlovic, 2023; Steinert, Hofmann, Leichsenring, & Kruse, 2015). Parsing this heterogeneity would enable identification of characteristics that make a person more likely to have a chronic condition and need mental health services. Previous work has focused on describing and explaining heterogeneity based on static attributes, such as symptom profiles, personality traits, and type of traumatic event (Campbell, Trachik, Goldberg, & Simpson, 2020, Campbell-Sills et al., 2022; Kelley et al., 2009; Thomas et al., 2014). However, no studies have examined *dynamic* attributes, such as the four mentioned before. Compared with static attributes, stress-symptom dynamics more directly reflect a critical source of heterogeneity in PTSD; namely, mechanisms that maintain a given person’s pathology over time. Identifying mechanisms that account for why one trauma-exposed person has a worse outcome than another is essential for effective and targeted treatment.

We propose a different approach that addresses gaps left by prior work. Specifically, we seek to understand heterogeneity in PTSD by characterizing dynamic rather than static characteristics. To do this, we examine *individual differences* in stress reactivity, stress generation, symptom persistence, and stress persistence for people exposed to trauma, in contrast to prior longitudinal research that has exclusively focused on the average effect of these dynamics. Our approach preserves heterogeneity in stress-symptom patterns which then allows us to determine which of these maintain trauma-related pathology and whether they could reflect interrelated mechanisms. Knowing the between-person covariation of within-person effects (i.e. dynamics) has important conceptual and practical implications. For example, if stress reactivity and stress generation are uncorrelated between people, this would imply they reflect two pathways to chronic PTSD, with many patients presenting with one primary temporal pattern or the other. In contrast, if stress reactivity and stress generation covary between people, this would suggest they reflect mechanisms that typically feed into one another and maintain PTSD via a ‘vicious cycle’. Because of the focus on average, within-person effects in nearly all prior longitudinal studies of PTSD, these fundamental properties of stress-symptom dynamics are unknown.

## Present study

In the present study, we aimed to provide new insights into mechanisms that maintain PTSD, and which give rise to a more chronic and severe course, by examining individual differences in stress reactivity, stress generation, symptom persistence, and stress persistence. We studied a sample of World Trade Center responders (*N* = 7,308) who were assessed 4–16 times spanning up to 20 years. The frequency of assessment and study duration is unprecedented in research on temporal associations between stress and PTSD symptoms, which have been mostly limited to two or three time points spanning less than 4 years. Our aims were to (1) identify within-person stress-symptom dynamics that characterize the long-term functioning of trauma-exposed people *on average*, (2) quantify the extent of *between-person variation* in these dynamics, (3) determine the *between-person covariation* in these dynamics, and (4) evaluate the validity and clinical utility of these dynamics by testing whether they predict the severity/chronicity of PTSD and mental health care service utilization.

## Methods

### Participants and procedures

Participants are members of the WTC Health Program Long Island Clinical Center of Excellence. Membership eligibility is determined by qualifying exposure during response to 9/11 terrorist attacks. Data for this study were drawn from annual visits to the health monitoring program. This is not a treatment-seeking sample, but rather the visits were akin to routine primary care check-ups. The program began monitoring responders in 2002 and has continued annual follow-up visits while maintaining open enrollment. To obtain reliable estimates of symptom dynamics, we only included participants with four or more visits (*N* = 7,308). On average, participants in our analytic sample had 8.8 visits. The distribution of visits per participant are reported in Supplementary Table S2.

Our sample was mostly White (74%; 4% Black/African American; 1% Asian; 5% other; 16% unknown/not reported) and male (90%), with an average age of 37.7 (*SD* = 8.16) on 9/11. Among responders who were assessed by clinical interview in the full sample from which our data were drawn, 17.7% met criteria for PTSD at some point after 9/11 (Bromet et al., 2016).

### Measures

#### PTSD symptoms

PTSD symptoms were self-reported at each visit using the trauma-specific version of the PTSD Checklist (PCL-17; Blanchard, Jones-Alexander, Buckley, & Forneris, 1996), with ratings referencing the WTC disaster. Participants rated past month DSM-IV symptoms on a scale from 1 (not at all) to 5 (extremely). All 17 items were summed for a total symptom severity score at each visit. Scores ranged from 17 to 85, with the average total symptom score across all visits and participants being 27.6 (*SD* = 13.06). A total of 21.1% of the sample reported clinically significant symptoms, on average, defined as scores over 35 (Terhakopian et al., 2008). Internal consistency (*ω*) for total PCL was 0.98 at the between-person level and 0.89 within-person.

#### Stressful life events

Life stress was self-reported at each visit using the Disaster Supplement of the Diagnostic Interview Schedule (Robins & Smith, 1983), which includes a checklist of 18 events (e.g. job loss, injury, arrest). Items were summed into a life stress composite. The average number of events reported each year was 1.26 (*SD* = 1.52). Endorsement rates of specific stressors are in Supplementary Table S2.

#### PTSD chronicity

Chronicity of PTSD symptoms was indexed by averaging each participants PCL total scale scores across the entire monitoring period.

#### Mental health care expenditures

The annual cost (in dollars) of participant’s mental health care services were obtained from their electronic medical health records. This included expenditures on psychiatric medications, psychotherapy, medication management, other office visits, and inpatient treatment billed to WTC Health Program for psychiatric disorders. These data were available for years 2018–2022.

### Analytic plan

Statistical models were estimated in R Version 4 (R Core Team, 2023) and Mplus Version 8 (Muthén & Muthén, 2020), in conjunction with the Mplus Automation package in R (Hallquist & Wiley, 2018). We used multi-level structural equation models (MSEMs) for our analyses. The MSEMs used Bayes estimator with non-informative priors that are default in Mplus (Asparouhov & Muthén, 2021). MSEM was used because it accounts for the nested structure of our data (i.e. time points within people) and it enables variables to be predictors and outcomes in the same model (i.e. bi-directional paths). Missing data was treated as missing at random and models were estimated using full information maximum likelihood techniques. Inferences were made from point estimates drawn from the posterior distribution and associated 95% credibility intervals. We considered parameter coefficients to be significantly different from zero if the 95% credibility interval did not include zero.

With MSEM, we decomposed the repeated measures of PTSD symptom severity and stressful events into between- and within-person latent variables. The between-person variables (i.e. intercepts) reflect individual differences in average levels of symptom severity and average stress exposure during the monitoring period. The within-person variables reflect fluctuations in symptoms and stress exposure away from a person’s average levels at a given monitoring visit.

**Figure 1.**
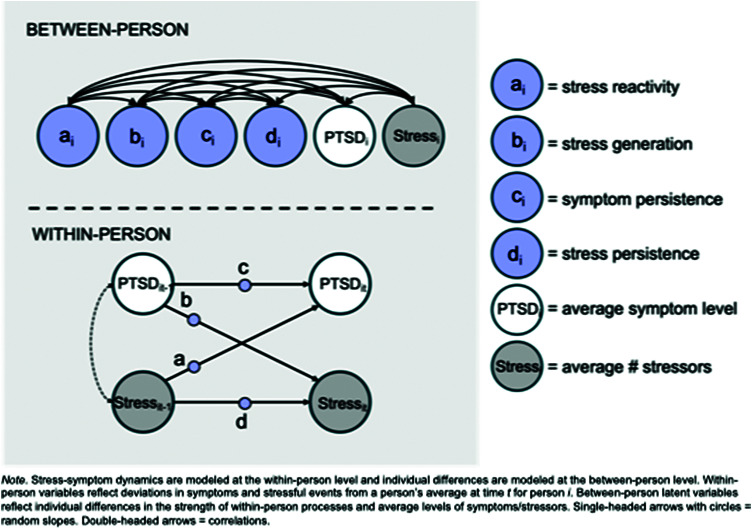
Multi-level structural equation model of stress-symptom dynamics. *Note:* Stress-symptom dynamics are modeled at the within-person level and individual differences are modeled at the between-person level. Within-person variables reflect deviations in symptoms and stressful events from a person’s average at time *t* for person *i.* Between-person latent variables reflect individual differences in the strength of within-person processes and average levels of symptoms/stressors. Single-headed arrows with circles = random slopes. Double-headed arrows = correlations.

The MSEM used for our analyses is depicted in Figure 1. Bidirectional processes linking symptoms and stress were estimated as random slopes at the within-person level. Stress reactivity was modeled by the cross-lagged association between stressful events_*t*−1_ → symptoms*_t_*, stress generation by the cross-lagged association between symptoms_*t*−1_ → stressful events*_t_*, symptom persistence by the autoregressive effect of symptoms_*t−*1_ → symptoms_t_, and stress persistence by the autoregressive effect of stress events_*t−*1_ → stress events*_t_.* Symptoms and stress at the same visit were allowed to covary. The fixed effects of these within-person processes reflect patterns that characterize functioning *on average.*

Random slopes were then estimated at the between-person level alongside the intercepts for symptom severity and stressful events. The random slopes capture *between-person variation* in the strength of within-person processes. All random intercepts were freely correlated. Correlations among the random slopes capture *between-person covariation* in processes. Uncorrelated slopes indicate they are relatively independent processes and people tend to present with one pattern or another (e.g. people are either more reactive *or* tend to generate stress) whereas correlated slopes suggest they are functionally related (e.g. people who are more reactive also generate more stress).

Finally, we evaluated the *clinical utility* of stress-symptom processes. To determine whether these processes contribute to the chronicity and severity of PTSD, we first examined between-person correlations among the random slopes and the PTSD symptom intercept (i.e. stable levels of symptom severity) within the MSEM framework. Then to increase the translational value of our results, we quantified clinical outcomes for people with varying levels of each mechanism. To do this, we first extracted individual-level within-person slope estimates. Slopes were estimated from the person-specific posterior distributions obtained as part of the within-person standardization procedure in Mplus (Schuurman et al., 2016). Next, we grouped people into deciles based on the strength of each stress-symptom slope. Because our interest was in evaluating outcomes for people with a high standing on these mechanisms, we focused on comparing outcomes for those in the top two deciles to those in the lower deciles. Namely, we compared the average PCL scores (i.e. chronicity) and total dollars spent on mental health services for people in upper and lower deciles of stress reactivity, stress generation, symptom persistence, and stress persistence.

## Results

Key parameters for the MSEM are in Table 1.

**Table 1.** Key parameters of multi-level structural equation model of stress-symptom dynamics

Average within-person slopes						
	Standardized *β*	95% CI	IQR			
Stress reactivity	.16*	(.11, .17)	12			
Stress generation	.03*	(.02, .03)	6			
Symptom persistence	.10*	(.09, .10)	12			
Stress persistence	.01	(−.01, .02)	7			
**Between-person correlations between slopes and intercepts**						
	Average symptom level	Average # stressors	Stress reactivity	Stress generation	Symptom persistence	Stress persistence
Average symptoms						
Average # stressors	.59*					
Stress reactivity	.27*	.10*				
Stress generation	−.03	.05*	.18*			
Symptom persistence	.08*	−.03	−.02	−.01		
Stress persistence	.11*	.19*	.31*	.49*	.11*	

*Note:* CI, credibility interval; IQR, interquartile range, * = credibility interval does not contain zero.

### Average stress-symptom dynamics

The average within-person slopes for stress reactivity, stress generation, and symptom persistence were significant. Stress reactivity was the strongest effect and stress generation was the weakest. Stress persistence was not significant. These results indicate that for people exposed to trauma, symptoms tend to worsen after major life stressors, worsening symptoms lead to more stressors than usual, and it often take months or even years to return to baseline functioning after a symptom flare-up.

### Individual variation and covariation of stress-symptom dynamics

**Figure 2.**
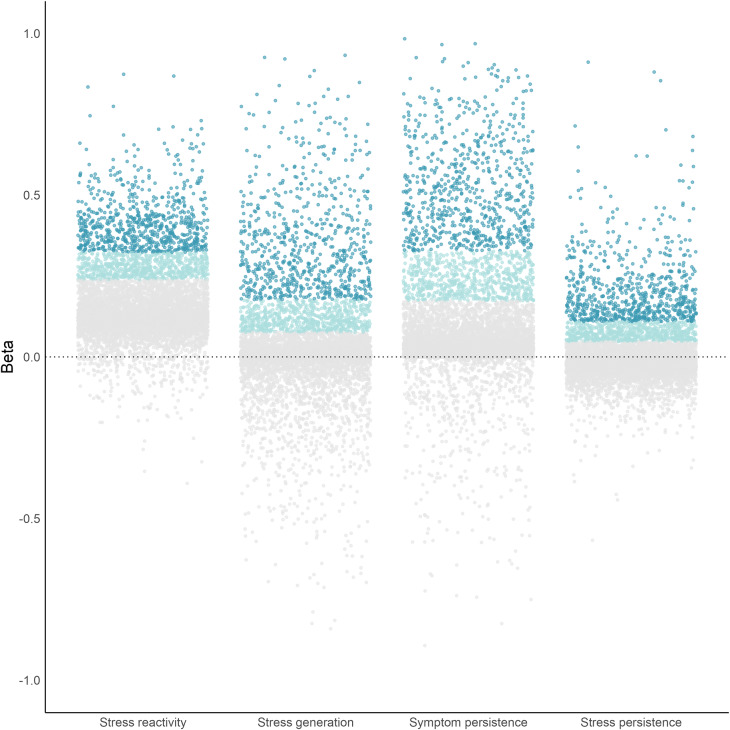
Individual differences in within-person stress-symptom dynamics. *Note:* Each point is an individual slope estimate. Stress reactivity is the individual’s average cross-lagged stress_*t−*1_ → symptom*_t_* slope; stress generation is the individual’s average cross-lagged symptom_*t−*1_ → stress*_t_* slope; symptom persistence is the individual’s average autoregressive symptom_*t−*1_ → symptom*_t_* slope; stress persistence is the individual’s average autoregressive stress_*t−*1_ → stress*_t_* slope.

There was significant variance in every random slope, with inter quartile ranges between 0.06 and 0.12 (model-estimated variance parameters are reported in Supplementary Table S3). Individual differences in the strength of stress-symptom dynamics are shown in Figure 2. Individual slope estimates ranged from strongly negative (minimum *β* = −.89) to strongly positive (maximum *β* = 0.98), although the majority of slopes were positive. This wide variability in the strength of stress-symptom dynamics highlights how the average within-person effects typically reported in longitudinal studies ignore considerable heterogeneity.

**Figure 3.**
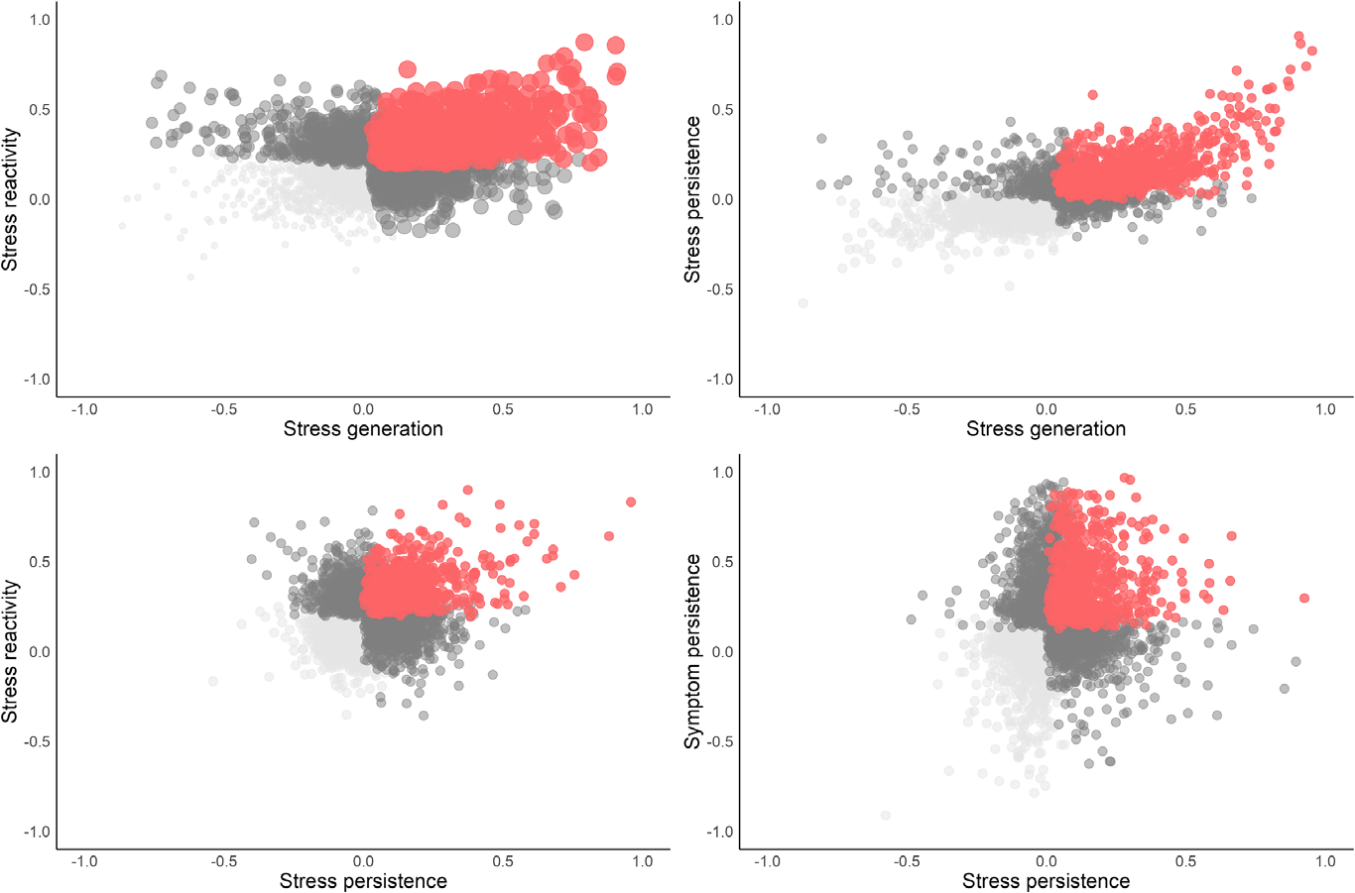
Between-person covariation of stress-symptom dynamics. *Note:* Each point is an individual slope estimate. The *x* and *y*-axes are standardized beta coefficients. Stress reactivity is the individual’s average cross-lagged stress_*t−*1_ → symptom*_t_* slope; stress generation is the individual’s average cross-lagged symptom_*t−*1_ → stress*_t_* slope; symptom persistence is the individual’s average autoregressive symptom_*t−*1_ → symptom*_t_* slope; stress persistence is the individual’s average autoregressive stress_*t−*1_ → stress*_t_* slope.

Correlations among random slopes indicate that there are related *and* independent processes. Stress reactivity was correlated with stress generation. Additionally, stress reactivity, stress generation, and symptom persistence were correlated with stress persistence. Scatterplots of individual slope estimates visualize the co-occurrence of processes in Figure 3. As shown in the figure, these processes co-occur across the sample, and there are people in the top deciles of each slope that incur a ‘double hit’ of interlocking dynamics. In contrast to these correlated processes, symptom persistence was not correlated with stress reactivity or stress generation despite all being significant, on average. These results reinforce that finding multiple, significant paths at the sample level does not necessarily mean those processes are typical of any given individual.

In sum, we found that people whose symptoms worsen after stressful events, and those whose symptoms lead to more stressors, also tend to experience more enduring stressors, whereas people who tend to have a slow return to baseline after symptom flare-ups are not particularly sensitive to stressful events and their symptoms do not lead to more stress.

### Chronicity and clinical burden associated with stress-symptom dynamics

After establishing there is significant variation in these processes, the final step of our analyses was to evaluate whether those individual differences are clinically meaningful. Starting with results in the MSEM (see Table 1), the stress reactivity and symptom persistence slopes were correlated with the symptom intercept (i.e. average levels of symptom severity).

**Figure 4(a).**
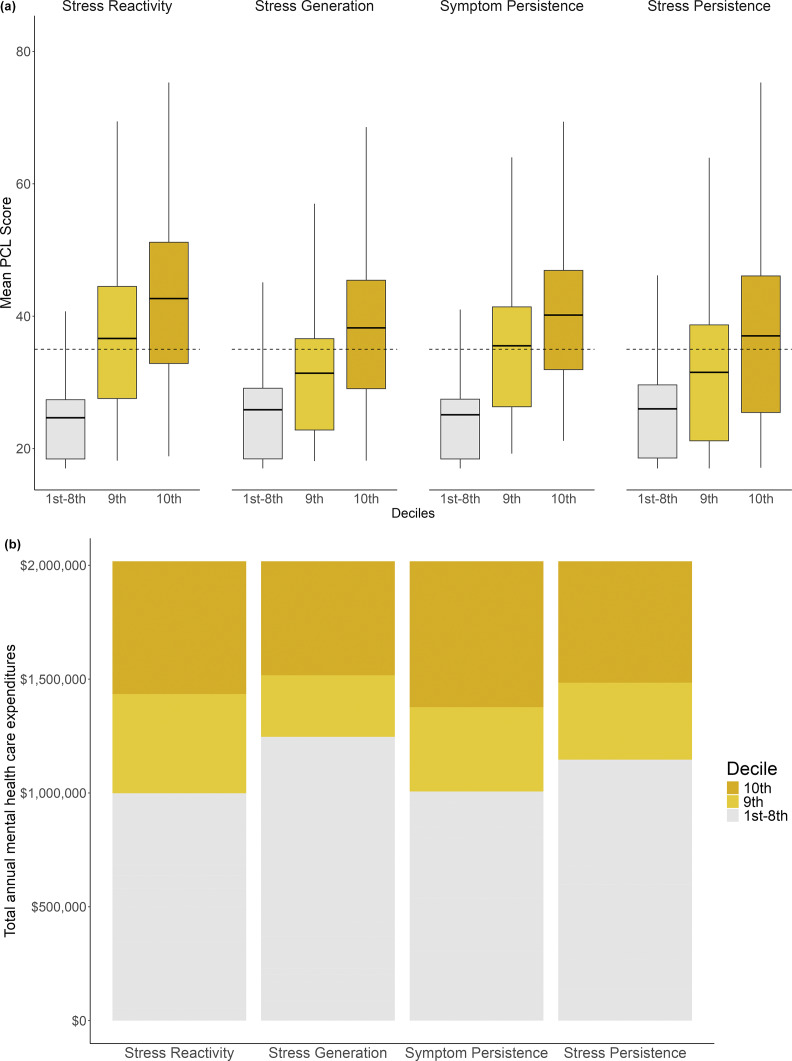
(a) Mean PCL scores for people at upper and lower deciles of stress-symptom dynamics. *Note:* Dotted line = threshold for clinically significant symptoms (i.e. PCL total score > 35; Terhakopian et al., 2008). The horizontal line in the box indicates mean level of PCL for that decile. (b) Mental health care expenditures for people at upper and lower deciles of stress-symptom dynamics. *Note:* Expenditures include cost of mental health care services and psychiatric medications.

Next, we examined associations between the four dynamics and chronicity of symptoms indexed by the observed, average total PCL score for people in each decile of slope estimates. People in the top two deciles of stress reactivity and symptom persistence, and top decile of stress generation and stress persistence, had clinically significant symptoms when averaged across the monitoring period. Chronicity of PTSD generally decreased in descending deciles of stress-symptom slopes, with people in most lower 8 deciles experiencing symptoms below cut-offs for clinical significance, on average. These results are shown in Figure 4a and reported in full in Supplementary Table S4.

Finally, we compared the annual expenditure on mental health services for people across deciles of stress reactivity, stress generation, symptom persistence, and stress persistence. These results are visualized in Figure 4b and reported in full in Supplementary Table S5. Individual differences in stress reactivity and symptom persistence had the most pronounced impact on service utilization; people in the top two deciles spent more per year than everyone in the respective lower 8 deciles *combined* ($1,019,029 vs. $998,596 for stress reactivity and $1,011,231 vs. $1,006,394 for symptom persistence). Put another way, mental health care for a person in the top decile of stress reactivity will cost over 10 times the amount that services cost for a person in the median decile ($812 vs. $78 per year), and services for those in the top decile of symptom persistence will cost over 15 times the amount than for people in the median decile ($884 vs. $56 per year). Likewise, although lower in absolute terms, services for people in the top deciles of stress generation and stress persistence cost 17% and 6.6% more than the median deciles, respectively.

Overall, these final analyses show that stress-symptom dynamics predict how severe a person’s symptoms will be over many years and how much mental health treatment they will need.

### Role of initial trauma exposure severity

In supplementary analyses, we examined the role of WTC exposures (e.g. lost someone, duration on site) in stress-symptom dynamics with two sets of analyses. A full list of exposure frequencies is in the Supplementary Materials. First, given evidence that trauma exposure severity largely dictates the course of PTSD (Boasso et al., 2015; Jakob et al., 2017), we tested whether stress-symptom dynamics predicted our clinical outcomes over and above exposure severity. The *R*^2^ change from hierarchical regression models with 10 WTC exposures in block one and the four stress-dynamics in block two showed that dynamics accounted for an additional 7% of the variance in mental health service expenditures (block one *R*^2^ = 0.01) and 29% of the variance in average PCL (block one *R*^2^ = 0.05).

Next, we tested whether exposure severity moderated stress-symptom dynamics. Two theories make predictions about the effect of exposures on these dynamics: stress sensitization theory predicts trauma increases stress reactivity and kindling/stress autonomy theories posit exposures increase symptom persistence. These analyses extend results from a prior study of this sample showing that exposure severity moderated stress reactivity over 2 years (Zvolensky et al., 2015a) by testing moderation over a much longer duration of time and for three additional stress-symptom dynamics. In these analyses, we regressed each stress-symptom dynamic on the 10 exposures in separate models, with the multiple *R* indexing moderation. Replicating prior work, we found that higher exposure severity associated with stronger stress reactivity (*R* = 0.10; *p* < 0.001). Additionally, exposure severity associated with stronger symptom persistence (*R* = 0.14; *p* < 0.001) and stress persistence (*R* = 0.10; *p* < 0.001), but not stress generation (*R* = 0.05; *p* = 0.295).

To summarize the role of WTC exposures, we showed that stress-symptom dynamics incrementally predict clinical outcomes over and above exposure severity and people who had more severe trauma tend to be reactive, have symptoms that persistent irrespective of major stressors, and have more enduring stress.

## Discussion

We sought to identify dynamics reflecting key psychological maintenance mechanisms that govern the long-term course of PTSD. Our results show that a clinically important source of heterogeneity in PTSD are individual differences in dynamics. Specifically, stress reactivity, stress generation, symptom persistence, and stress persistence may reflect distinct sets of mechanisms that account for why some people exposed to trauma go on to experience a more chronic course of PTSD and need more treatment than others.

First, we established stable stress-symptom dynamics that characterize the long-term symptom trajectories of trauma-exposed individuals, on average. Specifically, we found that PTSD symptoms tend to increase following stressful events, increases in symptoms tend to precipitate stressful events, and symptomatic episodes tend to have a slow return to baseline levels. These extend prior longitudinal studies that have been limited to following participants for much shorter intervals by showing these dynamics continue to be relevant decades after the original traumatic event.

It should be noted that our life events measure lacked some information needed for optimal tests of stress generation and stress persistence, which may partially account for these being the weakest effects. For stress generation, it is typically conceptualized as symptoms preceding dependent stressors (i.e. those that result in part from person’s behavior/characteristics) rather than independent stressors (i.e. those that are fateful and happen irrespective of a person’s actions) (Hammen, 1991), but we were unable to make this distinction with our measure. Meta-analytically, symptoms across diagnoses do indeed predict dependent events most strongly, but they also predict independent events (Rnic et al., 2023). These prior findings suggest there may be indirect ways that symptoms create stress, such as symptom-driven choices in environments that increase exposure to stressors or difficulties with actively avoiding stressors when especially symptomatic (Hammen, 2020; Rnic, 2023). Thus, it is likely our stress generation effects would have been stronger had we only included dependent events, but the relative strength of such direct and indirect generation paths related PTSD remains an open question. Likewise, for stress persistence, it is possible that significant effects would be found by focusing on events expected to penetrate multiple domains of life and lead to more stressors. In particular, chronic stressors may be especially likely to have this sort of permeating effect (Pearlin et al., 1997), but our measure only included discrete events. Research using a life events measure with more context, and which include chronic stressors, is needed to more comprehensively test stress generation and stress proliferation effects in PTSD.

There were significant and clinically meaningful individual differences in these core stress-symptom dynamics that allowed us to describe and understand heterogeneity in trauma-related pathology. Each of these dynamics can be interpreted as a PTSD phenotype that reflects different mechanisms. Patterns of covariation between people in these dynamic phenotypes bring clarity to how the underlying mechanisms may actually play out. This type of mechanistic information could not be gleaned from averaged, within-person effects that has been the focus of prior work. We clarified that although stress reactivity and stress persistence are both present on average, they are uncorrelated between people. This result indicates that these often reflect two distinct clinical phenotypes: trauma-exposed individuals who are especially sensitive to stress and those who tend to have a slow return to baseline after an episode. Such separable pathways, in turn, suggest that the underlying mechanisms operate relatively independently.

We also found evidence for mechanisms that may work in concert to maintain PTSD. Our results showed that people who are more reactive to stress also tend to generate stress, which implies their condition is maintained via a maladaptive, self-perpetuating feedback loop. For example, avoidant behaviors could erode social supports or lead to poor job performance (Breslau, Lucia, & Davis, 2004; Solomon & Mikulincer, 1990; Wang et al., 2021), or intrusive memories could lead to secondary depression and anxiety that compound impairments (Lawrence-Wood, Van Hooff, Baur, & McFarlane, 2016). Our findings also showed that people who experience more persistent stress are more reactive to stress, generate more stress, and have more persistent symptoms. The co-occurrence of these phenotypes may occur because exposure to chronically stressful environments makes it more difficult for people to recover from episodes (i.e. symptom persistence) or makes them more vulnerable to the effects of acute stressors (i.e. stress reactivity) (Cohen, Murphy, & Prather, 2019; McEwen, 2017). Another possibility is that having stronger reactions (i.e. stress reactivity) or more enduring symptoms (i.e. symptom persistence) makes it harder for a person to properly manage stressors, which then prolongs stressful situations (Thompson et al., 2018). Evidence for the interplay of stress persistence and stress-symptom processes lays the groundwork for future investigations that can home in on the mediating behaviors or circumstances that account for such indirect effects.

Exposure severity may also influence individual differences in stress-symptom dynamics. We found results consistent with two prominent theories of exposure-related mechanisms: consistent with stress sensitivity, exposure severity amplified the effects of stress on symptoms (i.e. stress reactivity) and consistent with kindling/stress autonomy, exposure severity related to a tendency to have symptoms that endure for years independently of major life events (i.e. symptom persistence). Additionally, although not posited by prevailing theory, we found that people exposed to more severe trauma tended to have more persistent stress. This could be because trauma creates conditions for more chronic stressors–for example, perhaps those with more severe exposures experienced more relationship strain after 9/11 (Hammock et al., 2019) or developed cognitive impairment (Clouston et al., 2022), problems which could in turn make stressors more likely for many years. By connecting initial trauma exposure to individual differences in these dynamics, our provide new support for long-hypothesized etiological mechanisms, and suggest some that may be underexplored.

### Clinical utility of stress-symptom dynamics and translation to practice

In our final analyses, we showed that mechanisms underlying variation in stress-symptom dynamics determine, in part, who will go on to have a more chronic and severe condition. There was evidence that all four dynamics we examined relate to clinical outcomes over and above the effects of WTC exposure severity. People in the top decile of the stress-symptom dynamics are at risk of an unremitting course of PTSD, with most people in these groups experiencing clinically significant symptoms on average across the monitoring period. Stress persistence and stress reactivity were most strongly related to the chronicity of PTSD, with clinically significant symptoms present in the top *two* deciles.

The more severe and recurrent symptoms experienced by people in the upper deciles of stress reactivity, stress generation, and symptom persistence translate into considerably higher need for mental health services. To put our results into perspective, if we assume that the proportion of total costs attributable to each decile of stress reactivity scales up to the United States population level, this group alone uses 36.5 billion dollars’ worth of mental health services every year (51% of the estimated $76.1 billion in annual, direct health care costs; Davis et al., 2022). Thus, variation in these psychological processes have real-world consequences.

Our results have actionable implications for improving the clinical management of PTSD in the future. Prevailing practices for treating PTSD are essentially one-size-fits-all despite its well-known heterogeneity (Benish, Imel, & Wampold, 2008; Cloitre, 2015), but it would be more effective to deliver interventions targeting the specific mechanisms driving a patient’s pathology. We advance this goal by providing a proof-of-concept for a mechanism-based method of classifying patients. From this starting point, future research can identify specific mechanisms related to each stress-symptom dynamic and develop treatments tailored to address them. If future research supports the clinical utility of these stress-symptom dynamics, and interventions can be designed around them, implementation would be relatively straight-forward. To accomplish this, a stressful life event checklist and the PCL could be regularly assessed at routine health care visits, entered into patient’s electronic records, and longitudinal patterns of stress and symptoms could be automatically analyzed to produce slope scores indexing levels of stress reactivity, stress generation, and symptom persistence. A learning health system could then provide data-driven, individualized intervention recommendations based on the patient’s slope scores updated at each visit. For example, when a patient with a pattern of high stress reactivity reports an uptick in stressors, this could trigger a recommendation for mindfulness-based stress reduction therapy (Polusny et al., 2015). If a patient high on stress generation *and* reactivity endorsed stressful events, this could prompt a brief assessment of PTSD-related behaviors that lead to stressors (e.g. behavioral avoidance, flashbacks) to then determine a treatment capable of reversing the vicious cycle (e.g. exposure therapy, cognitive processing therapy; Haagen, Smid, Knipscheer, & Kleber, 2015). A patient high on symptom persistence, on the other hand, may not be especially sensitive to stressors, but instead an uptick in symptoms could trigger a recommendation for a brief, trauma-focused intervention to prevent a prolonged episode (Sloan & Marx, 2019).

Additionally, given the link we identified between stressor persistence and stress-symptom dynamics, interventions that address environmental/situational factors may be an under-appreciated component of PTSD treatment. If our results are supported by further research, this suggests that connecting patients with community resources targeted to the legal, vocational, social, financial, or housing-related stressors they are experiencing could have direct and indirect effects on their symptoms. Thus, our findings take one step toward a mechanism-based approach to classifying patients, which in turn can eventually inform development of targeted interventions that would allow clinics to allocate resources more efficiently and ultimately steer the long-term trajectory of PTSD.

### Limitations

The contributions of our study must be qualified by some limitations. First, the sample may not be representative of all trauma-exposed populations. Demographically, the sample was mostly White and male. Given that the presentation and course of PTSD has been shown to differ across demographic groups (Asnaani & Hall-Clark, 2017; Reynolds et al., 2016; Tolin & Foa, 2008), the long-term symptom dynamics that we found may not extend to people of other races or genders. Furthermore, the prevalence of post-9/11 PTSD in this sample is higher than what has been reported among trauma-exposed individuals in general population surveys (Bromet et al., 2017). Despite these limits to generalizability, our results would likely generalize to other high-priority populations, like veterans, who have a similar demographic make-up (U.S. Census Bureau, 2019) and rates of PTSD (Dohrenwend et al., 2008) as our sample. Second, because life events were self-reported, it is unclear the extent to which the observed effects are influenced by biased appraisals of events or memory biases. This complicates interpretation of mechanisms because it is possible, for example, that stress reactivity actually reflects a tendency to recall more stressors when in a symptomatic state rather than a response to objective increases in stressors. Brief, semi-structured interviews or the reports of close others (e.g. relatives) could help to mitigate potential biases in future work. Such methods could also allow for separating the effects of subjective stress appraisals from objective events, each of which likely play a role in maintaining symptoms (Lazarus & Folkman, 1984). Third, the life event checklist was limited to relatively major events rather than more minor day-to-day stressors. Accumulating evidence supports the role of daily hassles in maintaining psychopathology, including PTSD (e.g. Short, Boffa, Clancy, & Schmidt, 2018), thus, it may be useful in clinical practice to collect more frequent assessments of relatively minor stressors alongside annual major event checklists. Research comparing the clinical utility of assessing major events versus daily hassles could help to determine an optimal assessment protocol for identifying at-risk patients.

## Conclusion

Our study showed that individual differences in stress-symptom dynamics can explain and predict heterogeneity in the long-term course of PTSD. We propose that these patterns of stress and symptoms can eventually inform the development of clinical protocols tailored to the psychological mechanisms maintaining a person’s problems. A mechanistically informed approach to classification and treatment can ultimately curb the chronicity and severity of PTSD and reduce economic burden on the individual and society.

## Supplementary material

Supplementary information, code, and data needed to reproduce our analyses, as well as full model output are on the Open Science Framework (https://osf.io/crx9y/).
